# Breastfeeding in women with HIV infection: A qualitative study of barriers and facilitators

**DOI:** 10.1371/journal.pone.0303788

**Published:** 2024-07-26

**Authors:** Aida I. Chaparro, Dieunane Formul, Stephanie Vasquez, Rosina Cianelli, Ivan A. Gonzalez, Gwendolyn Scott, Joseph P. De Santis

**Affiliations:** 1 Division of Infectious Disease and Immunology, University of Miami Miller School of Medicine Department of Pediatrics, Miami, FL, United States of America; 2 University of Miami School of Nursing and Health Studies Coral Gables, Coral Gables, FL, United States of America; Sefako Makgatho Health Sciences University, SOUTH AFRICA

## Abstract

**Background:**

Until recently, breastfeeding has been contraindicated for women living with HIV (WHIV) in the U.S. However, given the numerous health benefits of breastfeeding, recommendations have changed to support parental choice to breastfeed through shared decision-making. Although specific guidelines for managing the care of these women and their infants are not yet available, various approaches have been successful without infants acquiring HIV from their virologically suppressed mothers, thus, establishing breastfeeding as a viable option for the rising number of interested WHIV. This descriptive qualitative study aimed to identify factors influencing infant feeding choices decisions among WHIV in a multiethnic and multicultural population.

**Methods and findings:**

A qualitative description design was used. WHIV who had given birth within 6 months were recruited using purposeful sampling. Data were collected using a semistructured interview guide in the participant’s preferred language. Content analysis was used, and barriers and facilitators were separated and used to generate the themes and categories. In total, 20 participants were interviewed, and from these interviews, 11 barriers and 14 facilitators that influenced the decision to breastfeed were identified. Major barriers were related to the interference with daily activities, fear of transmission, lack of a standardized approach to education, and maternal concerns. Key facilitators included the benefits and advantages of breastmilk, access to more scientific research information on breastfeeding in the context of HIV, advice from a lactation consultant, emotional connection and attachment with the child, support from family and partners, empowering and supporting autonomy and decision-making about infant feeding, providing feeding choices, access to the lived experiences of women who have successfully breastfed their infants, and collaborative relationship with the physician and other healthcare providers.

**Conclusion:**

The study identified barriers and facilitators to breastfeeding among WHIV that may influence their infant feeding decision-making process. More research is needed to guide the standardization of institutional policies and develop strategies to support breastfeeding in this population.

## Introduction

Since the recognition that infants born to women with HIV (WHIV) can potentially acquire the infection through human milk, the World Health Organization (WHO) has recommended breastfeeding in resource-limited settings. In those areas, the lack of safe drinking water and availability of formula was associated with higher mortality rates for non-breastfed infants compared to lower rates among breastfed infants when antiretroviral treatment (ART) was available [1]. In contrast, recommendations from the WHO, the US Department of Health and Human Services (DHHS), the American Academy of Pediatrics (AAP), and the Centers for Disease Control and Prevention (CDC) have strongly recommended against breastfeeding for WHIV in the U.S. where replacement formula feeding is safe and accessible [1–3]. Acknowledging the numerous health benefits that breastfeeding provides for the infant and their mothers, recommendations against breastfeeding likely have increased the health and socioeconomic inequalities that already exist for many WHIV, regardless of the setting [4–6].

In the U.S., formula feeding has been predominant for decades; however, in recent years breastfeeding is gaining popularity, and its rates have been increasing [7–9]. Reports suggest a growing interest in breastfeeding among WHIV in the U.S., where the majority of pregnant WHIV are adherent to effective ART, and maintain virological suppression throughout pregnancy and at the time of delivery, resulting in few cases of mother-to-child HIV transmission [10–14].

Infant feeding recommendations for WHIV in resource-rich settings have changed recently, introducing recommendations without an absolute contraindication for breastfeeding and acknowledging the desire of some women to breastfeed [15–19]. In the U.S., recent guidelines from the Panel on Treatment of HIV in Pregnancy and Prevention of Perinatal Transmission, the DHHS, and the CDC, have opted for an approach that supports parental choice through shared decision-making with healthcare providers. While avoidance of breastfeeding remains the only option that fully eliminates the infant’s risk of HIV acquisition, any person who desires to breastfeed should be supported by the best strategy to minimize the risk of HIV transmission. Guidelines recommend that parents receive patient-centered, evidence-based counseling on infant feeding options to facilitate an informed decision [2, 3, 20].

Reassuring reports of infants being breastfed by their virologically suppressed mothers, while on appropriate neonatal antiretroviral (ARV) prophylaxis with zero HIV transmission rates have been emerging in Europe and North America. As detailed recommendations are not yet available, the approaches to managing the care of these WHIV and their infants vary widely; nevertheless, to date none of the breastfed infants have acquired HIV infection [21–24].

The majority of WHIV described in these published reports from North America were native to Africa and had breastfed previous children in their respective countries of origin as recommended by the WHO [21–22]. In contrast, the population of WHIV that we serve in Miami is multiethnic and multicultural, with mothers born both in and outside of the U.S., mainly in Latin America and Haiti. We also have a significant number of mothers with perinatally acquired HIV who may express an interest in breastfeeding their infants. The change in recommendations, with breastfeeding no longer being a contraindication for virologically suppressed WHIV, has not been addressed in our specific diverse population. We aimed to obtain a better understanding of the specific barriers and facilitators of breastfeeding from the qualitative perspectives of these WHIV.

## Methods

### Design and setting

A qualitative description design was used to guide the study. This design is used when little is known about the phenomenon under investigation, and the design allows a straightforward description of the phenomenon without excessive abstraction and interpretation of the data [25]. Qualitative description was the most appropriate design for the study based on the aim to explore barriers and facilitators of breastfeeding among WHIV.

The study was conducted at the University of Miami Pediatric Infectious Disease Clinic, which currently provides care to approximately 80 infants at risk for HIV infection per year and 60 children and adolescents diagnosed with HIV infection. Infants born to WHIV in Miami-Dade County are referred to this clinic for testing and management of their perinatal HIV exposure. Infants are followed from shortly after birth until definitive exclusion criteria for perinatal HIV infection are achieved, defined by the DHHS as two or more negative HIV virologic tests, with one negative test obtained at age ≥1 month and one at age ≥4 months [2].

### Participants and sampling

Eligible participants were approached at their initial infant visit at the Pediatric Infectious Diseases clinic by the first author. Participants were recruited using purposeful sampling, which aims to identify participants who have the most insight into the phenomenon under investigation [26]. Inclusion criteria were a) be a WHIV who has given birth to an infant within 6 months; b) be age 18 years or older; c) be able to speak English, Haitian-Creole, or Spanish; d) be able to provide informed consent to participate, e) have had an undetectable HIV RNA viral load (VL) throughout pregnancy and at delivery. Exclusion criteria were a) lack of legal guardianship of the infant, and b) have any medical or psychiatric contraindications for breastfeeding. Participants were recruited from December 15, 2022, to May 15, 2023. Written consent was obtained and stored separately from the participant’s data. Only the first author had access to the list that allowed the identification of the participants. This was essential to protect the privacy and confidentiality of the participants, vulnerable WHIV, who may experience HIV-related stigma. Before any data collection, Institutional Review Board (IRB) approval was obtained. (Study # 20220924)

### Data collection

Data were collected in the form of in-person interviews, using a semi-structured interview guide detailed in Table 1. Interviews were conducted in a private room in the above-mentioned clinic. Interviews were conducted by the second and third authors. Interviews were done in the participant’s preferred language, by qualified bilingual English-Spanish (third author) and English-Haitian Creole (second author) research team members and were audio-recorded and transcribed verbatim. Spanish and Haitian-Creole transcripts were translated into English by the above-mentioned qualified bilingual research team members. At least one other member of the research team who did not participate in the original transcription reviewed the interviews to ensure accuracy.

**Table 1:** Semi-structured interview guide questions.

Category of Interview Questions	Interview Question
**Interview Question**	**1) To try to make breastfeeding as safe as possible for women with this infection, some conditions are required for the mother:** a) Is fully adherent to antiretroviral therapy (ART). b) Has had undetectable viral loads (VLs) throughout pregnancy and maintains undetectable VLs during breastfeeding. c) Comes to the clinic and is closely monitored (every 1–2 months). 2) **The infant must** a) Remain on ART while breastfeeding. b) Be seen in the clinic and get tested often (every 1–2 months). c) Is exclusively breastfed (not supplemented with formula) **3) Knowing this, would you be interested in breastfeeding your infant, even though you are living with this condition?** a) Why or why not?
**Barriers.**	If you could safely breastfeed your baby, despite having this infection, • What do you think would be some barriers to you being able to do this?
**Facilitators.**	If you could safely breastfeed your baby, despite having this infection, • What would encourage you or support you to be able to do this?
**Services needed from doctors and nurses to support women with HIV infection who wish to breastfeed their infants.**	How could doctors and nurses help women with this condition who want to breastfeed their babies?
**Additional information needed.**	How could doctors and nurses provide information to women with this condition/infection who want to breastfeed their baby?

### Data analysis

Content analysis was conducted to identify major themes and categories emerging from the interviews [27]. The matrix approach was used to systematically reduce the data and identify themes and categories [28]. Data on barriers and facilitators, which served as the units of analysis, were extracted from the interview transcripts. Transcripts were read and reread by the last author to allow the identification of barriers and facilitators that were separated in the matrix to generate consistent themes and categories. The last author’s analysis was initially confirmed by the fourth author. It is important to note that the fourth and last author were not involved in data collection. Finally, all authors met to obtain consensus on the final themes, agreeing that saturation was reached and that findings were consistent with barriers and facilitators. The data were reduced, grouped, and coded, and names were created for each category that was grouped by barriers and facilitators.

Analysis of the categories allowed patterns to emerge, and exemplary quotes were chosen to illustrate and support the categories and themes under barriers and facilitators.

### Rigor

Rigor, also referred to as trustworthiness and viewed as the equivalent of reliability and validity in qualitative research, [29, 30] was ensured using several established strategies. The authors used Lincoln and Guba’s evaluative criteria to establish the trustworthiness of the study [30]. Credibility was established by providing thick, rich, illustrative quotes from the participants to ensure that their experiences were accurately presented. Additionally, credibility was enhanced as all the authors had prolonged engagement with the study data and the topic of HIV overall. To support the dependability of the findings, all authors except authors five and six participated in the final analysis to document intercoder agreement. Confirmability was important to include because all the authors have clinical and/or research experience with this population. To minimize researcher bias, data were analyzed by the last author, confirmed by the fourth author, and further confirmed by all authors. Any disagreements in coding were resolved by discussion until all authors agreed on the final themes. Finally, by providing the details of the participants and the general context of the study, researchers who may want to conduct this study with other WHIV on other sites, are supported in determining the transferability of the results to their communities.

## Results

A total of 20 WHIV participated in the study. Demographic characteristics are reported in an aggregate form in Table 2. Baseline demographic information revealed that participants were in their mid-20s to mid-40s (with a mean age of 32 years) and self-identified as African American (*n* = 7; 35%), Hispanic (*n* = 7; 35%), or Black Haitian (*n* = 6; 30%). Although the majority were born in the US (*n* = 12; 60%), eight (40%) were born in other countries, mainly in Latin America and Haiti, but all participants have been living in the U.S. for at least 7 years. Most spoke English as their main language, but three (15%) spoke only Spanish, and two (10%) only Haitian Creole. Most WHIV acquired HIV in adulthood; however, four participants (20%) were perinatally infected with HIV. All but 6 (*n* = 14; 70%) had more than one child, and of those, the majority had prior experience breastfeeding before HIV diagnosis was made. All participants had higher education (10 finished high school and 10 had at least some college experience). All these characteristics align with the demographics of WHIV in South Florida.

**Table 2:** Demographic characteristics of women with HIV.

Variable	Results	
Age	25–44 years (mean = 32.5 years)	
Race/Ethnicity	African American	7
	Black Haitian	6
	Hispanic	7
HIV Acquisition Mode	Behavioral	16
	Perinatal	4
Years since Diagnosis	Behavioral	2–16 years
	Perinatal	27–32 years
Education	High School	10
	College	10
Place of Birth	USA	12
	non-USA*	8
Language	English	15
	Spanish	3
	Haitian Creole	2
No. of Children	This is First Baby	6
	Have Other Children	14
	• 8 had prior breastfeeding experience	

*5 Latin America, 2 Haiti, 1 Portugal; all at least 7 years in USA

**Fig 1:**
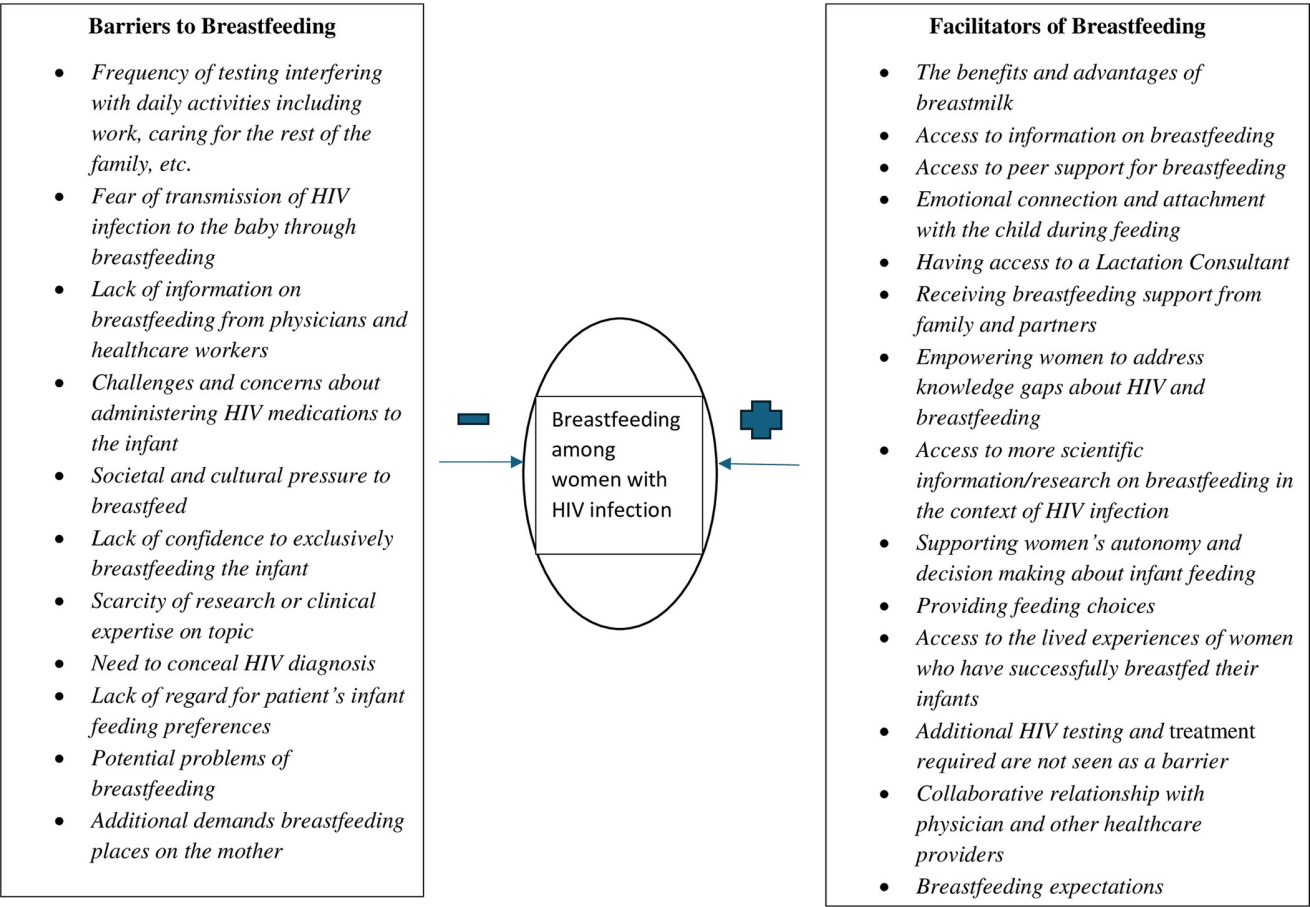
Barriers and facilitators of breastfeeding among women with HIV infection.

Participants provided thick and rich descriptions of the barriers and facilitators, and selected quotes from the participants are used to provide evidence for the categories identified from the data. Each category is supported by at least one participant quote. Fig 1 provides a summary of the categories of the barriers and facilitators of the decision to breastfeed among WHIV.

### Barriers to breastfeeding among women with HIV infection

Participants were able to identify 11 barriers that may interfere with the decision to breastfeed. Of these, four were deemed major categories because at least 25% (*n* = 5) of the participants endorsed these categories, while six were identified as minor categories because less than five participants endorsed these categories. Major categories included 1) *Frequency of testing interfering with daily activities including work and caring for the rest of the family*; 2) *Fear of transmission of HIV infection to the baby through breastfeeding*; 3) *Lack of information on breastfeeding from physicians and healthcare workers*; 4) *Challenges and concerns about administering HIV medications to the infant; and 5*) *Societal and cultural pressure to breastfeed*. Minor categories and the numbers (*n*) and percentages of participants endorsing the category included 1) *Lack of confidence to exclusively breastfeeding the infant*, (*n* = 3; 15%); 2) *Scarcity of research or clinical expertise on the topic*, (*n* = 3; 15%); 3) *Need to conceal HIV diagnosis*, (*n* = 3; 15%); 4) *Lack of regard for patient’s infant feeding preferences*, (*n* = 2; 10%); 5) *Potential problems of breastfeeding*, (*n* = 3; 15%)*; and 6) Additional demands breastfeeding places on the mother*, (*n* = 2; 10%). Additional quotes and quotes to support the minor categories also can be found in Table 3.

**Table 3:** Barriers to breastfeeding among women with HIV infection: Additional participant quotes.

Category (Major or Minor Category)	Participants’ Quotes
*Frequency of testing interfering with daily activities including work*, *caring for the rest of the family*, *etc*. • (Major category)	Yeah, the time and tests, I guess it’s a lot of times, like it’s hard enough to deal with the infection, you know. You know you gotta deal with other stuff for the baby to make sure that he doesn’t get it. So, I would just like, a lot, if you have to go through all that. I rather just give him formula. (Participant 1)
	Hmm.. No! (to breastfeeding). Well, the monitoring often…Yeah, that will be a disadvantage with all the appointments and then the shots. Then also the medicine. (It) is something that I would have to do myself, no one else can do it…I want to make sure I give her the medication, not anybody else. (Participant 17)
*Fear of transmission of HIV infection to the baby through breastfeeding* • (Major category)	I want (to breastfeed). I wish…I could breastfeed only because I would not want to feed my baby with formula, but rather milk that comes from me, and I am healthy, but, the HIV is in my body and if there is even a little chance that she could get it, that is just It’s an automatic no. Just for the fear of giving her any giving her this. I don’t want to. I don’t want to do that. I don’t have to. It would be stressful. We have that thought in your mind like you know, is that little chance, that at the next visit there is something there right?. . . It’s just a big thing. It’s to me it’s a big risk. Somebody else maybe thought would be smaller, for me is big! I would need my mind to be clear that my baby will be safe. I don’t think anybody would be able to help me unless it’s in my head, that I am sure that she’s going to be healthy, after every time she feeds, I will be thinking, you know, is (she) healthy? That is my dream that she is healthy. (Participant 11)
	…I know it’s everything has its’, I guess, risks. So, I would never want to feel like I put my son at risk of living with the condition that my other son (doesn’t have), simply because I just don’t think it’s fair to them… You know? You wanna do so good for your child, but at the same time, like you don’t know the amount of risk that you’re transmitting… I just feel like it would add some worry to my daily life, additional worries about transmission. Right? (Participant 12)
*Lack of information on breastfeeding from physicians and healthcare workers* • (Major category)	We should really have like more information when you go, because, like I, said the doctor, she was just like, “Oh, you can’t breastfeed. You’re not going to breastfeed.” I asked for a breast pump for I had, like inquired about it, they said, “I am not giving you a prescription for a breast pump because you can’t breastfeed.” It was vaguely talked about. It was very vague talking when I asked why I couldn’t breastfeed. So, as any smart person would do, I went online after, and I kind of like research the topic, but I guess it feels a little bit better if you speak to the person that’s taking care of you, if they want to tell me why I cannot breastfeed instead of going to the Internet. Having just a couple of doctors or nurses that kind of explain this to you a little bit better. Because I’m sure like there’s a lot of women with HIV (who) will like to know more… (Participant 1)
	I just don’t understand. I am right, you know, I take the medication, but I never asked…Why is it though, that even if being undetectable the baby could not breastfeed? I never asked, you know. Like I said, (with) that being the issue and my other daughter I didn’t give, you know my breastmilk, I was like, Okay, well, it’s not gonna be hard for me to give her the formula, but (was) something that has been in my mind, and I just never ask(ed)… In in my head I’m thinking, “Oh, I’m not gonna do it, I know she’s gonna be healthy, and I’m not gonna do it (breastfeed).” But I never asked. You know, I never asked, and it always is in my mind due to me taking my medication. (Participant 6)
*Challenges and concerns about administering HIV medications to the infant* • (Major category)	I would not do it…for the simple fact that I hate taking the medicine. And I would hate to put my baby through that if she doesn’t have to. (Participant 11)
	Because the baby must take the medications. . .just (the) long-term exposure to the medication…Just the baby being on medication for a long time. It is ok for me to be on medications since I already have to take it, but not the baby. (Participant 18)
*Societal and cultural pressure to breastfeed* • (Major category)	The only thing I can say is like, or when I was speaking to someone and she asked me about (breastfeeding), of course I do want to experience that…That’s something I always wanted to experience, but I couldn’t…and it’s just hurtful. The things I have to hear from my family (because I can’t breastfeed) …I hope, like hopefully. someone that’s going to do that or the same thing as me, they don’t have to go through it because of all the research that’s going on. Hopefully that the next one could do so. (Participant 2)
	I think they will not support it…Because in the Haitian community if you are found to be HIV positive you are stigmatized. The way they think about the condition, and their views on the disease, would cause them not to support breastfeeding, to discourage it, because of the stigma associated with it…They will only want me to bottle-feed the baby and nothing else. (Participant 18)
*Lack of confidence to exclusively breastfeeding the infant* • (Minor category)	I would have done it even with the diagnosis because if you do have some protocols that give you peace of mind. If it was just that the protocol, I would have done it. But that thing about exclusive breastfeeding. I’m not sure, because you also don’t know if you’re gonna produce enough milk, or at least enough for the baby. We don’t know how our body is gonna react. If it’s gonna work correctly or not. So, while having these protocols you can try to do it and if then you realize that it’s not working then you stop. If it is not only exclusive breastfeeding and you are able to also give formula if you need. If that’s the case I will do it…It is that it’s really not that easy. It is not as easy as it sounds and it not always works out. (Participant 20)
*Scarcity of research or clinical expertise on topic* • (Minor category)	Honestly, I know it sounds bad, but I (would) prefer other people try it first and check it. Because if it works for other people, I know it will work for me. Because I’ve been undetectable for years, and I’m still undetectable, and I’ve already experienced the whole making sure she takes her medication on time… As long as (I am) on top of that, because I’m already adjusted to giving her the medication. But I think I gave her a medication longer than she was supposed to take it. But I’m already adjusted to it to stuff like that. I think I wouldn’t mind. I just wanna make sure if it works for other people, and is there’s a sister that says, “Look, I tried it, and it works.” It’s like, “Okay, I’m okay.” (Participant 15)
*Need to conceal HIV diagnosis* • (Minor category)	Yes, and I had to make things up (reasons for not breastfeeding). Because it is not easy to explain the real reason and I cannot always say the truth, obviously. So, it is better to just make things up. I would definitely be questioned, because not only that I have to go to the visits with the specialist, but also, she has regular visits to the pediatrician, for vaccines, and so. . . . So that means that I have to go out to the doctor every 1 to 2 months and it’s just a lot. So, then there will be a lot of questions, like you know, “What’s wrong with the baby? Does she have something? You know, like, too much going on, too much question(s)… It’s like, “Why (are) you in the hospital every month?” So, then you would have to have now have an explanation…because then they will wonder what’s going on with the baby. (Participant 20)
*Lack of regard for patient’s infant feeding preferences* • (Minor category)	If they say they don’t want to breastfeed, (it) is fine. I don’t think it should be force(d) for women to go to all that step(s), depending on their home situation they could decide… Like, you asked me if I was willing to sit down and talk, and if I didn’t want, it was fine too. So (giving) people the option, yeah, and the option for them to do (it: breastfeeding), because, first of all, people could be hurting. You see, every time you guys, doctors, nurses come in the room with me I’m a little nervous, It’s a flash back from the diagnosis. (Participant 5)
*Potential problems of breastfeeding* • (Minor category)	So, I believe that if it’s done in a safe way, there will not be any disadvantage. The only disadvantage will be, that it could happen, is that the mom does not produce enough milk. And the other one will be that sometimes depending on the nutrition that the mom is getting some babies will have more colic or gas. But this will be an effort that we have to do to maintain our nutrition under control while breastfeeding. (Participant 20)

### Frequency of testing interfering with daily activities including work and caring for the rest of the family

The most common barrier to breastfeeding reported by eleven (55%) of the participants was related to how frequent testing for the breastfed infant might interfere with the participants’ other daily activities. Participant 20 summarized how additional medical appointments for HIV testing would impact her daily activities:

If mom has other kids or if she’s working and has other responsibilities, it will be very hard to remember all those things when you have a newborn baby. I believe that part will be complicated, to remember all the appointments, all the checkups, to remember about giving the medication. That’s where I see that it could fail because during those first days, you have too many things on your mind….

### Fear of transmission of HIV infection to the baby through breastfeeding

This category was the second most reported category, with nine (45%) participants expressing concern that the infant might acquire HIV infection through breastfeeding. This fear of HIV transmission is summarized by Participant 4:

The only thing that would be a barrier for me would be fear of the baby becoming infected with the virus, nothing else. I don’t mind coming (for medical appointments). I don’t mind staying here for a long time or coming often. If they tell me I have to do that, if the doctor said that, I will do it because they’re caring for me and the baby…

### Lack of information on breastfeeding from physicians and healthcare workers

This category, reported by six (30%) of the participants, was the first category that was not focused on the mother or infant. In this category, participants noted the lack of information about breastfeeding from physicians or other healthcare workers who provided care at any point during pregnancy. Participant 14 summarized how this lack of information influenced the decision to bottle-feed her infant:

Probably, you know, like the counselor letting them know that, even though I do have this problem (HIV infection), you can still breastfeed. Because no one, never the whole pregnancy, all three of them, nobody never mentioned that. Like if I’m taking my medicine properly and I’m undetectable, maybe I could breastfeed. The doctor could have said that, I mean everybody needs to know that is an option… if more information was out, I think more people would do it. Because if I’m healthy what will stop me from having healthy milk for my baby?

### Challenges and concerns about administering HIV medications to the infant

Five participants (25%) reported the need to administer HIV medications to the infant to reduce the risk of transmission of HIV through breastmilk might influence the decision to breastfeed. Participant 16 spoke at length on this topic:

Like now for example I’m gonna stop giving the medication to the baby. But in that case (breastfeeding), I will have to give something that she really doesn’t need, something that would be concerning to me, give a medication for such a young baby, and I could avoid that by using formula instead of breastfeeding. And for what I understand, that’s what will have to happen now. But if in the future if there is a way to breastfeed without having to give any medications to the baby, I will be the first one in the list…So my main concern it’s actually the medications, not the visits, just the medications. What if the baby develops an allergy to the medication? So, then I will have to give another medication and then another one? And those are the things that someone doesn’t know because it is just not known. But if you would not have to give any medication, I think there will be no disadvantage. So, my concern is the medications both the ones that you give to the mother and therefore go through the breast milk to the baby, as well as the medications that you give to the baby itself.

### Societal and cultural pressure to breastfeed

Five participants (25%) reported that social/cultural pressure to breastfeed was a barrier. Perhaps the pressures placed on these women were intended to encourage breastfeeding, but this pressure often resulted in the decision not to breastfeed. Participant 11 summarized the pressure to breastfeed:

I’m not really sure (about others in my culture), because the only people that were aware of my condition were my mom and my sisters and my brothers—close, close family. So, I really wouldn’t know about the rest. You do get judged a lot; that’s about it. Everybody’s gonna judge…They always want you to breastfeed. They ask you a million times. ‘Why not? Why not?’ And you can’t reply with anything because you get judged for not doing it (breastfeeding).

### Facilitators of breastfeeding among women with HIV infection

Participants were able to identify 14 facilitators that may promote breastfeeding among WHIV. Of these, thirteen were deemed major categories because at least 25% (*n* = 5) of the participants endorsed these categories, while one category was identified as a minor category because less than five participants endorsed these categories. The major facilitators were as follow: 1) *The benefits and advantages of breastmilk*; 2) *Access to information on* WHIV *breastfeeding*; 3) *Access to peer support for breastfeeding*; 4) *Emotional connection and attachment with the child during feeding*; 5) *Having access to a Lactation Consultant*; 6) *Receiving breastfeeding support from family and partners*; 7) *Empowering women to address knowledge gaps about HIV and breastfeeding*; 8) *Access to more scientific information/research on breastfeeding in the context of HIV infection*; 9) *Supporting women’s autonomy and decision making about infant feeding*; 10) *Providing feeding choices*; 11) *Access to the lived experiences of women who have successfully breastfed their infants*; 12) *Additional HIV testing and* treatment *required are not seen as a barrier*; and 13) *Collaborative relationship with physician and other healthcare providers*. One minor facilitator was identified; three participants (15%) endorsed the category of *Breastfeeding expectations*. Additional quotes to support the major and minor categories can be found in Table 4.

**Table 4:** Facilitators of breastfeeding among women with HIV infection: Additional participant quotes.

Category (Major or Minor Category)	Participants’ Quotes
*The benefits and advantages of breastmilk* • (Major category)	And it (breastfeeding) will be much easier like, as I said to you. Running up, getting up, making the milk, warm up the formula, one of the bottles. It takes a couple of minutes…Yeah, you know? Breastfeeding will make me more anti. . .less anxious, more calm? Definitely my call, breastfeeding for sure. I think it would be much easier, and I just roll over and take the breast out to her instead of they’re screaming the whole time, and I’m running, giving up. I’m like super tired getting up going to the kitchen. Since I have her bottles prepared, but even then, still it’s still a shock to the brain. especially if your mother, who’s been through something. . .(takes a deep breath). It makes it even a little bit worse…Your anxiety level is sky high. Your blood pressure goes up pretty fast. So, there you are being woken up suddenly, and the baby is crying and running, trying to hurry up and make a bottle, especially being a new mom. The baby is crying, you’re like, “Oh, my God!” You feel sad because the baby is crying. We want to feed them as fast as possible. (Participant 7)
	On top of that breastfeeding gives the baby the weight that the baby’s gonna need. Nutrients that the baby needs on top of the nutrients in formula…the add(ed) nutrients… Yeah, we’ll have a healthier baby. And also, it’s like, formula cause these other problems that they don’t have to experience. My baby has a lot of side works, has complications with feeding, so seeing her in pain, because I am not breastfeeding. . . you know, I hope that this makes sense. (Participant 15).
*Access to information on WHIV breastfeeding* • (Major category)	They should have pamphlets about breastfeeding, and they should have, pamphlets about HIV and breastfeeding, and why not, or why you should breastfeed. You know they’re gonna say you should (or) you shouldn’t, okay fine. Make a pamphlet for it, or you know a website, something…. I think for sure, talk to me first, it’s better. I always think that like…face-to-face communication is way better than because, like you can get to ask why, why not. Or maybe they can give you the pamphlet first read it, and then speak to somebody. Well, I think both (are) kind of like necessary, so you can take it home, read it when you analyze it, whatever, and then come back next time, and ask questions. Well, maybe like a first meeting class or something, because you know, they don’t teach you all I guess maybe if I was breastfeeding, they could have taught me at the hospital, like you know how to latch, a nurse could watch you and stuff just to make it like easier. Also. like breast pumping methods, and like how to store, you know, like women freeze their milk and stuff, and it’s like good for years and stuff. so those type of classes might help, or a class that helps you with latching and pumping and stuff. (Participant 1)
*Access to peer support for breastfeeding* • (Major category)	Also, you want the support, you will want to lean on other people going through the same experience. You know? It’s difficult for everyone. How could they (the physicians) help on paper, they know the numbers and everything, but not the motion of it? It’s different. It sounds like when it’s someone that is going through it with me and lived the other things that we had, (it’s) better.(Participant 10)
*Emotional connection and attachment with the child during feeding* • (Major category)	When women do breastfeed(ing), it bring(s) the baby a little bit closer. It’s attachment. (Participant 6)
	You will have a better connection with your baby. The bonding, thing that is a good experience, and all of this. (Participant 16)
*Having access to a Lactation Consultant* (Major category)	A lactation specialist would be good… Individually you can ask questions like personal questions. (Participant 3)
	So this would be like a one on one consultation (with a lactation consultant), right?. . .Yes, because it is for people who don’t really know how it works, like having someone explain it to them to show them, it will make it easier for them. (Participant 9)
*Receiving breastfeeding support from family and partners* • (Major category)	Yes, because if they (family members) don’t know that there are ways that you can do breastfeed(ing)…, they would think she’s going to get it (HIV infection) and would be concerned about that…If I explained it, then probably not. But if without the knowledge of knowing that, then you know, they will be all concerned. I mean if this is about breastfeeding, I will just ask my sister, since she did it already… I would say that you should surround yourself with people that will be supportive and will give encouragement as opposed to people who would just be negative about it. (Participant 9)
*Empowering women to address knowledge gaps about HIV and breastfeeding* • (Major category)	I think we should do it throughout all the pregnancy…in order to make the woman comfortable. I think they should always…do it throughout the whole pregnancy so that way they can feel much more comfortable, because if they learn after pregnancy right after giving (birth), you would be iffy about it. But if …I get the working on that information throughout the pregnancy, …they can make their own choice to go in on research, some more on their own to practice. (Participant 2)
*Access to more scientific information/research on breastfeeding in the context of HIV infection* • (Major category)	It’s like, you never know what tomorrow brings, the brain, science, and medication, and the whole medical field changes so quickly, you know. New advances come every day and challenge things that are in the works. So you know, if a little bit more research is done about breastmilk, HIV, and the effects on the child. Then I wouldn’t mind breastfeeding. You know what, for this pregnancy the formula seems to work for me and (my baby). So I wouldn’t mind breastfeeding, like I said, It’s a way to bond. Both options have their pros and cons themselves. So, you know? Nothing is ever perfect but If they do a little bit more studies on it and like it’s told to me by the professional doctor that okay, you could breastfeed. You need to have the virus controlled and he is not gonna catch it. Then I would. (Participant 1)
*Supporting women’s autonomy and decision making about infant feeding* • (Major category)	Well, everyone has their own opinion, their own thoughts, everyone has the right to think differently, and I do respect other people’s opinions. Everyone knows what they’re doing… Yes, I would say that it’s important not to be judgmental, that every woman should decide if they want or not to breastfeed, that everyone should know what is better for their kids and it’s a personal decision. (Participant 13)
*Providing feeding choices* • (Major category)	I just want to experience it to see what it feels like, because, seeing everybody else do it. I’m like. I wish I could at least experience it, to be able to feel that bond. (Participant 9)
	…If you breastfeed you feel like more like other people within your community. Yeah, you would feel less isolated. (Participant 10)
*Access to the lived experiences of women who have successfully breastfed their infants* • (Major category)	Yes, that helps keeping people with hope. Like, “Okay, okay, she’s done it. You know? I want to hear her story, especially from other women with similar, with the same cases that want to breastfeed, that had breastfed. It would help a lot, you know? Like success stories. Yeah, it would bring a piece of mind to some women that do want to breastfeed while having the condition (HIV infection)…Anything like a pamphlet would be nice. It would be helpful, very helpful. But it’s always better word of mouth. Yeah, directly, yeah, from somebody that did it, because anybody can write a pamphlet, you have to have the disease to know (it) right?. . . Not from someone breastfeeding that doesn’t have the disease and is telling somebody that does. (Participant 11)
*Additional HIV testing and treatment required are not seen as a barrier* • (Major category)	Extra medications not a concern (facilitator). That doesn’t bother me, because I have to take medication. So, it’s like when I take mine, baby takes those, you know? It’s like a the same time thing. So, I’m gonna make sure that she, my main thought, is always gonna be making sure my baby’s okay. So, I’m gonna make sure. I remember to get for her. So, then it also triggers me. Okay, I got also take mine. So, it’s like a parental aspect of it. I guess… Look if I have to come to the doctor every month, I don’t mind, because that’s one day I get to call a lot of work, ok?. Okay, I get an extra day off. (Participant 15)
*Collaborative relationship with physician and other healthcare providers* • (Major category)	I mean, going to your doctor’s appointments. It’s very important that you don’t miss your doctor’s appointments, I mean, they’re the ones that is gonna be there regardless when you need to help. I mean the only people that’s gonna probably go through that process with you, and how you will be is your doctor and the social worker. I’m like, she helped me with everything, “Oh, you have a dental appointment,” or “Oh, do you have refills for your medication, or how are you feeling?” She’ll say, “Oh you know a holiday will come up.” (You) have a sense of community, you know, yeah. And you know, if I need anything, or when I go to go see him, I’ll stop by and see her, or you know… But I think is very important. But I mean, like I said, our issue is maybe like people with cancer and maybe like other conditions. And I think that’s important. (Participant 6)
*Breastfeeding expectations* • (Minor category)	I imagine culturally, it’s expected of you within the African American culture to breastfeed. So, people might not ask questions. (Participant 8)
	Yes, I have been asked…Well, yeah, I forgot when, it was someone talking to my partner, so he says, “Oh, she had a C section, and I gave her some pain medication, and she don’t wanna, you know, to breastfeed because the medication that they gave her for pain could do something to the baby.” And they were, “Oh, yeah, that makes sense.” (Participant 19)

### The benefits and advantages of breastmilk

This was the most common facilitator of breastfeeding reported by all 20 participants. All participants were aware that breastmilk was better for the infant when compared to formula. Participants 12 and 16 summarized the benefits and advantages of breastmilk:

I’ve always understood that it (breastmilk) carries antibodies for the baby, nutrition that the mother gives off. So, I feel like those are the advantages of the baby being able (to be breastfeed) versus a kid who’s only formula fed and not breasts (breastfed).

The benefit of health, growth and everything else. The same thing that you tell anyone that does not have HIV, to breastfeed. All the good and the beautiful things that they tell moms that don’t have HIV so that she will breastfeed. If you were a mom and you will know for sure that you’re not gonna give HIV to your baby, obviously taking good care of yourself, I will bet you 100% sure that you will breastfeed your baby. The benefits are the same and they’re known. (The) baby would be stronger, healthier… Oh, and also baby will have a very good immune system because you pass those cells through the breast milk.

### Access to information on WHIV breastfeeding

Sixteen participants (80%) reported that access to information on breastfeeding might facilitate the woman’s decision to breastfeed her infant. Participants 2 and 4 provided information on not only the importance of access to information but suggestions on how to best provide this information:

Well, doctors [and Nurse practitioners] …should…give more access to resources where they can, so they can feel comfortable…. Let them (women) know how there’re some other countries that moms are breastfeeding and their baby turned out to be ok. And maybe give out flyers about that. So, having additional information from the doctors, but not just verbally, but also having like resources available like…pamphlets and written (material).

If they (physicians) did research and they sat down with me and give information about the findings, I would want to know what research they did, what they found out, and what their recommendations are before I decide.

### Access to peer support for breastfeeding

In addition to information, thirteen participants (65%) wanted access to peer support to encourage breastfeeding. Having this peer support in the form of a breastfeeding support group could be a way to facilitate breastfeeding, as reported by Participant 16:

It is really good to have a support group, you realize that you are not the only person that is there. To know that you’re not the only one going through that uncertainty, and having all those questions in your mind, is always helpful. To know that you’re not alone…

### Emotional connection and attachment with the child during feeding

Eleven participants (55%) reported that breastfeeding would provide an emotional connection that would promote attachment with their babies during feedings. This potential facilitator was noted by Participant 12:

I guess bonding. I feel like my son. I don’t know if this is true, but like when I hold my son like, I feel like he feels the sensation of my breast, and he’ll wanna like, you know (breastfeed) go and have it, obviously I’m wearing clothes, but I’m just like okay, I can’t so I’ll give him the nipple of the bottle, so I feel like maybe (breastfeeding might be) a bigger connection with your infant…

### Having access to a Lactation Consultant

Ten participants (50%) reported that having access to a Lactation Consultant who was familiar with the breastfeeding guidelines for WHIV might facilitate breastfeeding. Women who had other children often noted how a Lactation Consultant was not available to them before the change in feeding options for WHIV. Participant 16 described how the Lactation Consultant might promote the decision to breastfeed:

…But I think that a lactation specialist will be very important. If there is a lactation group, that they have as leader a specialist and that is available at least once a week wherever you go for your control., it would be great. So, you can have kind of a routine checkup with that person and make sure that everything is OK. That will give you lots of reassurance…

### Receiving breastfeeding support from family and partners

Nine (45%) participants believed that support from family and partners might facilitate breastfeeding. Knowing that breastfeeding is a significant commitment, participants needed support from their families and partners to encourage them during this process. Participant 10 described how she could be supported during breastfeeding:

I have a lot of support (from the family). You have more support there. Yeah, especially with help of my husband. Yes, you know? I guess that having him (for support), is like something that would help. He helps with the medication. I have somebody to come with me to the appointments.

### Empowering women to address knowledge gaps about HIV and breastfeeding

Eight participants (40%) reported that empowering women to address knowledge gaps about breastfeeding in the context of HIV infection served as a facilitator to encourage breastfeeding. This category was described by Participant 20:

So, I would like to have one visit (with) just doctor and me and definitely way before the delivery. I will say at least a month or two before delivering. I have to receive all the information ahead of time to review it. So, when the time to have the baby comes, I will be already clear about my decision and what I would like to do. The earlier the better, so you can be well prepared and take time to decide and find out what is what you want to do. If I have all the information from doctors and nurses and all the orientation and education from people that is dedicated to this, I could make up my mind.

### Access to more scientific information/research on breastfeeding in the context of HIV infection

Six participants (30%) believed that breastfeeding among WHIV could be facilitated if provided with the latest scientific information and research on the topic. Many of the participants reported that having access to the information readily available might encourage breastfeeding as noted by Participant 7:

It’s really nice to see that they’re working on (doing more research) helping mothers (with HIV infection) to breastfeed. It’s like when I first found out about my situation (HIV diagnosis), I never thought that today (it) would be possible for me to have a baby. Naturally, I was like shocked when I first saw this, but now, things are changing…you know? Doctors are really in there working (doing research) and something.

### Supporting women’s autonomy and decision-making about infant feeding

Five participants (25%) noted that the decision to breastfeed among WHIV was based on supporting the women’s autonomy in making this decision. Participants wanted to be provided with as much information as possible and wanted to be respected for the decision made. Participant 16 succinctly summarized this category:

I think that is important that they make us feel that we can participate in the decisions… So anyway, if we need to make (a) decision about what’s best for our families it’s important to be educated correctly.

### Providing feeding choices

Closely linked with the previous category; five participants (25%) believed that the option to breastfeed their infants provided feeding choices that were not previously available. Knowing that WHIV were instructed on exclusive bottle feeding to prevent HIV transmission, having two feeding choices may be a facilitator of breastfeeding according to Participant 6:

So, I think some women, too, with this situation (HIV infection) go through their ups and downs, and I think it gives them an open door, like a sign a relief, that they’re able to do the same thing (to breastfeed) as a regular woman as they didn’t have an infection. So, you know, if that was an option, I think it’ll be great.

### Access to the “lived experiences” of women who have successfully breastfed their infants

Five participants (25%) noted that they might be more likely to consider breastfeeding their infants if they could have access to WHIV who have breastfed their infants without them acquiring the infection. This “lived experience” seemed to instill trust and might be a way to encourage more WHIV to breastfeed according to Participant 2:

Have the women that (are) HIV positive that (are) still breastfeeding come out and talk to other women. There can be a one on one, or it could be a group setting. And then also having what you’re referring to, like a peer kind of person. Yeah, someone that we can relate to, that can come out and speak to us, either one or one or group setting…talk with someone with the same condition (HIV infection) that is breastfeeding, and this child turned out to be perfectly fine. So that would be great too… you know, like giving us more like research.

### Additional HIV testing and treatment required are not seen as a barrier

During the interviews, participants were provided information on the HIV testing requirements and the medication regimen that the mother and the infant would need to adhere to, to prevent HIV transmission during breastfeeding. Despite the additional appointments that would be necessary, five participants (25%) did not perceive these extra appointments as a barrier. As summarized by Participant 9, the extra appointments might facilitate the decision to breastfeed:

No, not me. I wouldn’t have a problem with that at all, because I had to come more often than that when I was pregnant. So, it’s okay. I mean it’s really no big deal about that… I mean coming to the doctor a lot. I’ve been doing that since when I was 20 years old, since then all the way into now. So, it’s not really a barrier for me, and then medication that’s something I know too, so, it’s like it’s not really a bother to me…. Because I found out in July 2016, and I started medicine, I believe in August, and then in September, I was undetectable. And I have been undetectable ever since… Oh, no, I have been doing this for so long, that nothing will beat me!

### Collaborative relationship with physicians and other healthcare providers

The final major category was reported by five (25%) of the participants. In this category, participants noted that breastfeeding could be facilitated if the women could establish collaborative relationships with personnel providing their care. As mentioned in previous categories, participants wanted to be supported in their decisions and wanted to be provided with feeding choices. These would promote the collaborative relationship, which then may promote breastfeeding, as described by Participant 7:

Oh, nurses and doctors, they can just basically just be there for us (to) answer any questions and concerns that we have and give us you know, their knowledge on the subject…When you have your appointment and we’re going to talk about, you know whether you (are going) to have a baby or you have one. They could just inform you about any new information…for someone who have just had their first baby in my position, I think, just by going to your doctor they should verbally let you know about why you can breastfeed… and you know, if it’s safe, if it gets to be 100% safe where you don’t have to take meds, then I think they should put it on the news… when I met my current doctor, you know she’s the one who has me now over 6 years. So, she’s the one who say (said), “Girl, you could have a baby. Just stay undetected (undetectable). You know these new meds are coming out. Eventually we’re gonna switch you into something better.” …So, I was like, “Wow! Yeah!”

## Discussion

The findings of this study identified various barriers and facilitators that strongly influence breastfeeding practices among WHIV in South Florida, U.S. These results significantly contribute to the established knowledge base, particularly considering the limited studies available in North America. Furthermore, this study allows for additional discussion and reflection on how to support informed decision-making regarding breastfeeding among WHIV.

The most common barrier reported by study participants was the interference of frequent testing with their daily activities. This finding highlights the significance of providing support and implementing strategies to assist mothers with HIV in effectively breastfeeding their infants, adhering to testing protocols, and managing their everyday responsibilities. Existing literature suggests that family members and partners play crucial roles in promoting breastfeeding and supporting infant care [31, 32]. Family-centered breastfeeding education programs can decrease the burden on mothers, especially concerning the challenges associated with HIV infant testing. Moreover, implementing a system enabling healthcare workers to conduct laboratory tests for infants at home would eliminate the need for mothers to arrange transportation and endure waiting times for infant testing, thus enhancing convenience and accessibility, as well as avoiding disruption of an already busy routine. The establishment of home-based services, such as doulas, lactation specialists, peer mentors, and psychosocial workers, could assist women to navigate the challenges of complying with complex protocols and appears to be a valuable strategy to better support breastfeeding among WHIV [33, 34].

Furthermore, the fear of transmitting HIV infection to their infants through breastfeeding emerged as the second most reported barrier. Participants expressed concerns about the potential risk of transmission, despite the low likelihood of this occurrence in the context of viral suppression. This fear can significantly discourage some mothers from breastfeeding, despite their willingness to comply with medical appointments and recommendations. Fear of HIV transmission has been reported in multiple studies as a major reason for avoidance or early discontinuation of breastfeeding [32, 35–38]. This finding highlights the crucial need for physicians to provide evidence-based information with tangible examples demonstrating the increasing numbers of WHIV who have breastfed their infants without transmitting the infection. A multidisciplinary patient-centered approach that provides psychosocial and mental health services as well as peer counseling and access to lived experiences could provide further reassurance to address these fears and support mothers in making well-informed decisions regarding breastfeeding [32–35, 38, 39].

Another barrier identified by participants was the inadequate provision of breastfeeding information by physicians and healthcare workers; the existing literature confirmed this barrier [31–35, 39]. Participants expressed frustration with providers failure to directly address feeding options, including breastfeeding, during their pregnancy. The absence of explicit information regarding the possibility of breastfeeding for WHIV left some participants feeling uncertain about their feeding choices. Additionally, challenges and concerns associated with administering HIV medications to the infant emerged as an important barrier to breastfeeding. Participants expressed worries about having to give antiretroviral medications to their infants for extended periods and the potential risks and adverse effects associated with such. The delivery of accurate and updated information by healthcare providers, especially when it is tailored to specific patient needs and concerns, has been shown to reduce some of the concerns expressed by the mothers and to facilitate shared decision-making between the mothers and their providers [19, 32–35, 39].

In addition, societal and cultural pressures to breastfeed were also reported as a barrier by a subset of participants. While these pressures may be well-intentioned to promote breastfeeding, they may interfere with the decision-making process for WHIV. Participants expressed being questioned and judged by others for not breastfeeding, which added an emotional burden to an already complex decision. This is often highlighted in the literature describing the detrimental impact that feelings of scrutiny regarding feeding choices have on mothers with HIV [36–38]. As social pressure may extend beyond inner/close family circles, the provision of breastfeeding education that includes significant others, extended relatives, and friends is crucial [31–35, 39].

The results of this study also revealed several factors that facilitate breastfeeding among WHIV. The most significant facilitator reported by participants was the awareness of the benefits and advantages of breastmilk compared to formula. All participants recognized that breastfeeding improves infant health and boosts the infant’s immune system, and identified this as a major motivation to breastfeed. This finding aligns with existing literature indicating that well-informed mothers who considered breastfeeding as valuable and have a positive attitude toward it, are more likely to initiate and sustain breastfeeding [35, 39, 40]. Furthermore, emotional connection and attachment with the infant during feedings were also identified as significant facilitators of breastfeeding. Participants expressed that breastfeeding created a special bond with their infants and fostered a deeper emotional connection. It is well established that attachment and responsive infant care are crucial for the child’s neurophysiological, physical, and emotional growth and development [5, 41]. Therefore, it is important to implement programs that provide education in effective breastfeeding techniques as well as psychosocial support to the emotional needs of the mother, facilitating a positive breastfeeding experience [37, 38, 41–43].

Additionally, support from family and partners was considered essential for successful breastfeeding among participants. The mothers emphasized the importance of having a supportive network during the breastfeeding experience that provides not only emotional but also practical support with simple tasks, such as diaper changes, bathing, and burping the infant after feedings. The implementation of family-centered breastfeeding education that prepares partners and the extended family for active support can promote breastfeeding and effectively alleviate the burden on breastfeeding mothers [31, 32, 36, 44].

Another important facilitator identified by participants is the need for qualified and skilled healthcare professionals who educate, support, and positively influence their attitudes toward breastfeeding, which is congruent with existing literature [35, 39, 40, 44]. As some mothers may not have the confidence to assert themselves in terms of seeking more information from their healthcare providers, a more systematic approach to infant feeding counseling, that prioritizes transparency in choices and options, encourages an open discussion of concerns, and respects and supports decisions, could empower mothers, and potentially increase breastfeeding rates among this population [32–36, 39]. Participants also emphasized the importance of having lactation consultants, who are knowledgeable about breastfeeding in the setting of HIV. Lactation experts spending time observing the mother and infant during breastfeeding can provide technical and emotional support, identify potential problems, and address questions and concerns [34, 41, 42, 45]. This aligns with existing literature that breastfeeding interventions that integrate the expertise of lactation consultants contribute to higher rates and longer duration of breastfeeding [41, 42].

Lastly, most participants identified peer support as a significant facilitator for breastfeeding. Support groups are valuable sources of encouragement, validation, and opportunities for sharing common experiences and knowledge. Participants emphasized the importance to connect and relate to other women facing similar challenges, and expressed that having access to lived experiences mothers with HIV who successfully breastfed their infants would be particularly reassuring. For WHIV, peer support and support groups can serve as valuable resources, providing a safe space to discuss concerns and fears, share experiences, and promote informed decision-making regarding infant feeding practices [37, 43]. Since peer support was reported as such an important factor with only limited applicable peer groups available, the creation and training of a peer network is crucial for the successful implementation of breastfeeding programs for WLHIV.

## Conclusion

In conclusion, our study has identified multiple barriers and facilitators to breastfeeding among WHIV, which may significantly influence their decision-making process. The findings contribute to the limited available research and provide crucial considerations for healthcare professionals. This particularly diverse multiethnic and multicultural study population differs from the previously studied populations and therefore, offers a unique opportunity for further insight into this multifaceted topic.

Healthcare professionals have an essential role to play in empowering WHIV by addressing these barriers through accurate information dissemination and supportive counseling. Moreover, incorporating family and peer support is crucial in assisting these mothers in making informed choices that balance the potential benefits of breastfeeding with the need to minimize HIV transmission risk.

Programs and interventions aimed at promoting and supporting breastfeeding among WHIV must take into consideration the specific breastfeeding facilitators as well as the barriers described in the study. The need for more comprehensive care for WHIV regarding infant feeding remains evident, starting by implementing breastfeeding training to healthcare providers and multidisciplinary team members to ensure that they are knowledgeable and skilled in breastfeeding support. The study also highlights the importance of recognizing the autonomy of the mother in the decision-making process and encourages a personalized approach when developing care plans to enhance the overall outcomes for mothers and their infants.

Future research should aim to develop and evaluate tailored interventions and healthcare strategies specifically designed to address the barriers that may interfere with breastfeeding and implement the facilitator strategies to support and guide WHIV in their decision-making process regarding infant feeding.

## Supporting information

S1 Data (XLSX)
